# Modulation of Nrf2 expression by targeting i-motif DNA

**DOI:** 10.1038/s42004-024-01387-w

**Published:** 2025-01-06

**Authors:** E. F. Warner, D. Guneri, M. A. O’Connell, C. J. MacDonald, Z. A. E. Waller

**Affiliations:** 1 School of Chemistry, Pharmacy and Pharmacology, University of East Anglia, Norwich, Norfolk UK; 2 UCL School of Pharmacy, London, UK

**Keywords:** Screening, DNA, DNA

## Abstract

Nuclear factor (erythroid-derived 2)-like 2 (Nrf2) is a key regulator of cell detoxification, which maintains homoeostasis in healthy cells and promotes chemoresistance in cancer cells. Controlling the expression of this transcription factor is therefore of great interest. There are many compounds that have been shown to induce *Nrf2* expression, but ligands that can inhibit Nrf2 are scant. Herein we characterise an i-motif-forming sequence downstream of the *Nrf2* promoter, which we hypothesised may regulate the expression of the gene. The Nrf2 i-motif was found to be stable at near-physiological conditions. We identified small molecule ligands that interact with this i-motif structure and one significantly upregulated Nrf2 mRNA expression, and one ligand reduced Nrf2 mRNA expression in human cancer cells. This is the first example of controlling the promoter of *Nrf2* by targeting DNA structures and offers an alternative mode of action for the development of compounds to improve the chemotherapeutic responsiveness of existing treatments for cancer.

## Abstract (web summary)

Nuclear factor (erythroid-derived 2)-like 2 (Nrf2) is a transcription factor that regulates the expression of proteins with various intracellular functions, however, the ligands that directly control the Nrf2 promoter are underexplored. Here, the authors identify small molecule ligands that regulate the expression of Nrf2 by targeting a newly identified i-motif-forming sequence in the Nrf2 promoter.

## Introduction

Nuclear factor (erythroid-derived 2)-like 2 (Nrf2) is a transcription factor that regulates the expression of proteins serving a wide range of intracellular functions, such as detoxification and reduction of reactive oxygen species, which have anti-inflammatory and cytoprotective properties^1^. Global mapping of binding sites for Nrf2 has validated over 240 genes as targets for Nrf2 in cell survival response^2^. Nrf2 has been shown to have potential as a chemopreventive by effects on detoxification, response to oxidative stress and inhibiting DNA damage and inflammation^3^. It is also involved in tumour promotion through not only chemoresistance but in many of the hallmarks of cancer and potential drug resistance in tumours^4^. The prevalence of Nrf2 in the hallmarks of cancer strongly suggests that targeting this transcription factor could be a good therapeutic approach^5^.

Under normal conditions, Nrf2 is ubiquitinated and degraded under the regulation of Kelch-like ECH-associated protein 1 (Keap1). Upon activation, such as by an inflammatory or oxidative stimulus, Nrf2 accumulates in the nucleus and subsequently activates transcription through binding to the antioxidant response element (ARE) in the *Nrf2* promoter region alongside small Maf proteins^1^, regulating the expression of cytoprotective enzymes, such as haem oxygenase-1 (HO-1) and NAD(P)H dehydrogenase quinone 1 (NQO1). Many ligands have been shown to disrupt the interaction of Nrf2/Keap1^6^, to the best of our knowledge none have reported the therapeutic potential of controlling the promoter of *Nrf2* directly. It has been shown that ligands that target DNA sequences capable of forming secondary structures (e.g. G-quadruplex and i-motif) present in the promoter regions of several genes, can modulate gene expression^7–9^. Given its dualistic nature and pathophysiological importance, Nrf2 is therefore an interesting target for this mechanism.

We have previously identified a G-quadruplex forming sequence on the leading strand within the *Nrf2* promoter sequence^10^. Others have shown that a G-quadruplex in the Nrf2 mRNA 5’ untranslated region regulates de novo Nrf2 protein translation under oxidative stress^11,12^. Despite this, the putative i-motif structure in the promoter region of the gene, which would form opposite the DNA G-quadruplex, has not been investigated at all. i-Motifs are non-canonical quadruplex secondary DNA structures defined as two parallel stranded duplexes held in an antiparallel manner by the intercalation of hemi-protonated cytosine–cytosine^+^ base pairs^13^. i-Motifs require hemi-protonation of cytosines to stabilise in vitro, however it has been demonstrated that this can occur at neutral pH^14^ in conditions such as molecular crowding^15^. The presence of i-motif structures in cells has been shown by use of fluorescent antibodies and in-cell NMR experiments^16–18^, as well as whole genome methods^19^. This has increased the importance of understanding the functional role of these DNA structures and how these can be modulated for therapeutic purposes, particularly for targeting proteins where intervention cannot be achieved by other means^20^.

Here we characterise the i-motif forming sequence within the promoter region of the *Nrf2* gene by use of circular dichroism (CD) spectroscopy, UV spectroscopy, and ^1^H NMR. Further, we present ligands identified through a high-throughput fluorescent indicator displacement (FID) assay and determine the effect of these ligands on carcinoma cell viability and their bioactivity on *Nrf2* gene expression.

## Results and Discussion

### *Nrf2* promoter sequence forms i-motif at near physiological pH conditions

The *Nrf2* promoter sequence has been shown to contain a G-quadruplex at −606 base pairs from the transcription start site and was identified as a potential quadruplex forming sequence^10^. The sequence, which we will refer to as Nrf2G, denoting it as the G-rich sequence 5′-GGG-AAG-GGA-GCA-AGG-GCG-GGA-GGG-3′, is close to putative transcription factor binding sites in the *Nrf2* promoter^21^. To examine the i-motif we used the reverse complement, termed Nrf2C: 5’-CCC-TCC-CGC-CCT-TGC-TCC-CTTC-CC-3’.

**Fig. 1:**
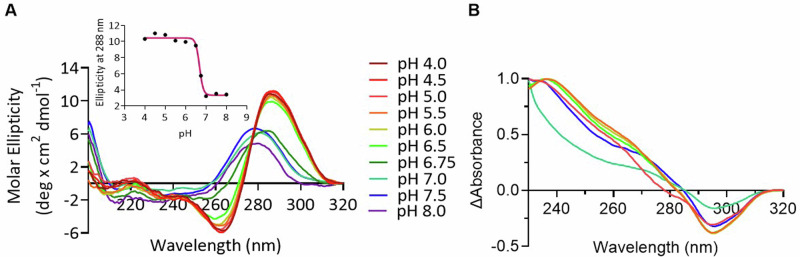
Biophysical Characterisation of the i-motif forming sequence from *Nrf2.* **A** Circular dichroism spectra of Nrf2 (10 µM DNA) in buffer containing 10 mM sodium cacodylate, 100 mM potassium chloride, at pH 4.0-8.0. Transitional pH (pH_*T*_) was determined using ellipticity at 288 nm (inset). **B** Thermal difference spectra of Nrf2 (2.5 µM DNA) in buffer containing 10 mM NaCaco, 100 mM KCl, at pH 5.0, 5.5, 6.0, 6.5, 7.0 and 7.5.

Circular dichroism (CD) was utilised to determine the transitional pH (pH_*T*_) of the oligonucleotide sequence upstream of the *Nrf2* transcription start site (Fig. 1A). Samples of Nrf2C (10 µM DNA) were annealed in buffer containing 10 mM sodium cacodylate, 100 mM potassium chloride, at pH 4.0-8.0. i-Motif structure was determined by previously characterised parameters: a positive peak at ~288 nm and a negative peak at ~255 nm^22^. The pH_*T*_ of this i-motif forming Nrf2 sequence was determined to be pH 6.7 (Fig. 1A). The sequence is a relatively short i-motif forming sequence [5’-CCC-TCC-CGC-CCT-TGC-TCC-CTT-CCC-3’] with a tract length of three cytosines. Compared to other i-motif forming sequences, this is relatively stable for a tract length of three cytosines. For example, the human telomeric sequence has a pH_*T*_ of 6.3^23^, and the i-motif from the promoter region of the hras gene a pH_*T*_ of 5.9^24^.

UV spectroscopy was utilised to determine the thermal stability (*T*_m_*)* at pH 5.0, 5.5, 6.0, 6.5, 7.0 and 7.5 (Supplementary Fig. S1 and Supplementary Table S1). The *T*_m_ of the structure at pH 6.5 was 27 ± 0.4 °C, making these conditions suitable for further investigation at room temperature. There were no significant differences between melting and annealing experiments, indicating fast folding and unfolding kinetics. This is in contrast to many i-motif forming sequences examined previously under the same conditions, where hysteresis was observed^14,23^. UV spectroscopy was further utilised to determine the thermal difference spectra (TDS) of Nrf2 (Fig. 1B). This measurement was used to characterise the dominant folded species of the Nrf2 secondary structures across a pH range. At pH 5.0-6.5, the TDS of Nrf2 demonstrated a positive peak at 240 nm and a negative peak at 295 nm, characteristic of folded i-motif structure^25^. Even at pH 7.0 the spectrum was consistent with i-motif, but the appearance was significantly different to the other pHs examined.

^1^H NMR was used to verify the formation of this i-motif structure at pH 6.5 (Supplementary Fig. S2), where imino proton signals can be observed at 15.5 ppm at 298 K, characteristic of the C^+^–C base pairs in i-motif ^26^. Further imino signals present at 12.5-13 ppm indicate additional Watson-Crick base pairing interactions^27,28^. These additional base pairing interactions may provide further stability to the structure, and are likely the reason for the elevated transitional pH compared to the C-tract length. There are actually five tracts of cytosines in the sequence [5’-CCCTCCCGCCCTTGCTCCCTTCCC-3’] separated by potential loops of 1, 1, 5 and 2 nucleotides between each C-tract from the 5’- to the 3’- end. This means there is potential for more than one conformation. The presence of Watson-Crick imino protons indicates that there are some G-C base pairs (as there are no adenines in the sequence). A potential structure to accommodate these base pairs could involve a long central middle loop of eight nucleotides: 5’-CCCTCCC**GCCCTTGCT**CCCTTCCC-3’ which would have the potential to form two CG base pairs. Interestingly, there are no TT base pairs observed. Given the presence of many thymines in the sequence and recent evidence to suggest that i-motifs are often stabilised by the presence of thymines contributing to TT base pairs in the loops^29,30^, this is surprising. Nevertheless, this conformational flexibility between potential different structures offers opportunity for switching by ligands, as previously demonstrated in the i-motif forming sequences from KRAS and Bcl2^31,32^.

Taken together, our experiments demonstrated the Nrf2 i-motif was stable in vitro at near-neutral pH. Previous work using in-cell NMR has shown that i-motif forming sequences in cells are more stable than those in vitro^16^, owing in part to molecular crowding^15^. This indicated that the Nrf2 i-motif forming sequence in the promoter region had the potential for targeting with small molecule ligands.

### i-Motif binding ligands identified using a fluorescent indicator displacement assay

Using a previously developed thiazole orange (TO) based FID assay^33^, a screening experiment of 1584 drugs present in the National Cancer Institute (NCI) Developmental Therapeutics Program (DTP) was conducted to identify ligands that bind to Nrf2 at pH 6.5. We identified 11 ligands that displaced thiazole orange by greater than 20% at 2.5 µM (Table 1) and the concentration required for 50% displacement (DC_50_) for each of these compounds was determined using the same method. We note that the results from compound 202386 were outside the normal expected range for error – consistent with difficulties with solubility under the conditions used in the experiments. These 11 ligands were taken forward to explore their cellular bioactivity in a cancer cell-line.

**Table 1:** Ligands identified from FID screening

Compound	NSC	*D*_TO_ (%)	DC_50_ (µM)
	71795	59 ± 0.5	3.9 ± 1.0
	317003	43 ± 3.8	3.4 ± 0.5
	63842	47 ± 3.6	1.5 ± 0.1
	143491	34 ± 5.7	4.9 ± 1.5
	73735	36 ± 0.4	2.3 ± 0.1
	134674	32 ± 2.9	3.2 ± 0.1
	202386	27 ± 20.3	>20
	61642	26 ± 8.7	12 ± 3.4
	354844	25 ± 1.4	6.3 ± 0.6
	36758	22 ± 0.7	16 ± 2.7
	300289	20 ± 4.8	13 ± 3.0

Structures of hit compounds from the FID assay with 0.5 µM Nrf2 in 10 mM sodium cacodylate, 100 mM potassium chloride at pH 6.5, % displacement of TO from the screening at 2.5 µM (*D*_TO_) and the concentration required for 50% displacement of TO (DC_50_).

### Modulation of Nrf2 in human colon carcinoma cells

**Fig. 2:**
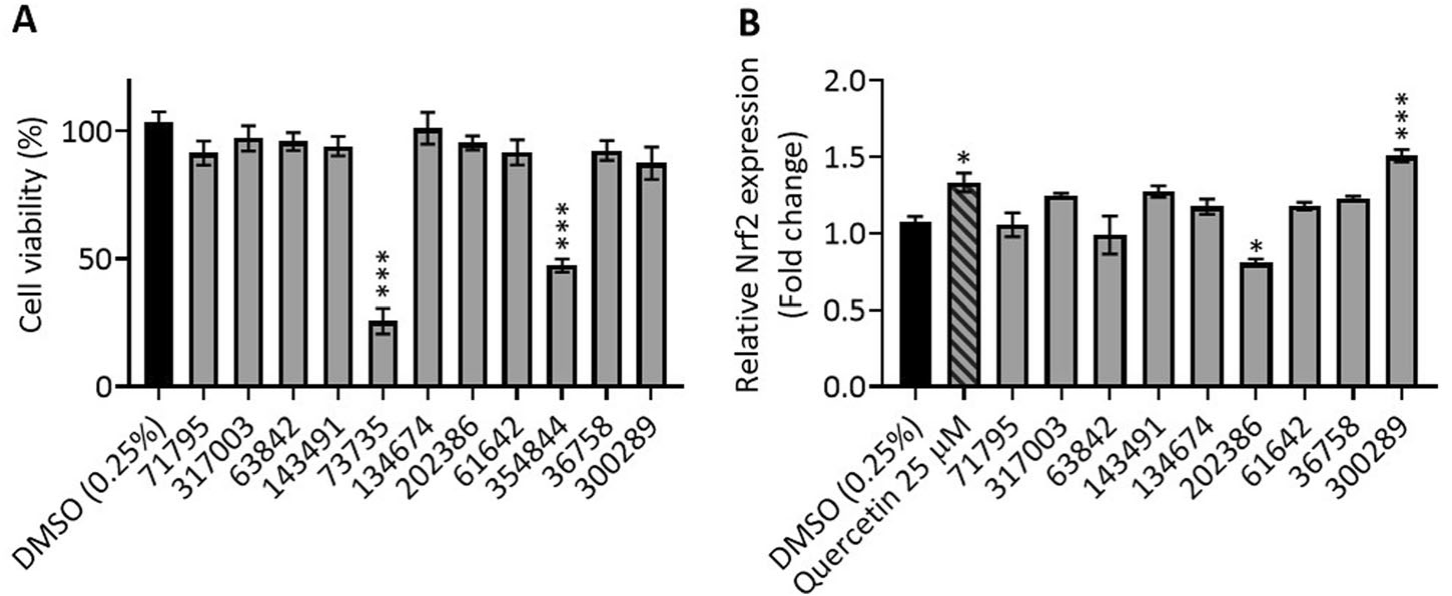
Bioactivity of Nrf2 i-motif-binding ligands. **A** Effect of Nrf2 i-motif-binding ligands on HCT-116 cell viability. Cells in monolayer were treated with 2.5 µM of each ligand (Table 2), 0.25% DMSO, or media only for 24 h prior to the addition of MTS reagent for 4 h. Columns represent absorbance values presented as a percentage of an untreated (media only) control (mean ± SEM, *n* = 4). **B** Effect of Nrf2 i-motif binding ligands on Nrf2 mRNA expression in HCT-116 cells. Cells in monolayer were treated with 2.5 µM of each ligand (Table 1; grey bars), 0.25% DMSO (vehicle control; black bar), quercetin (positive control; stiped bar) or media only for 4 h prior to RNA extraction, reverse transcription and RT-qPCR. Ct values were normalised to a GAPDH control and columns represent the fold change relative to an untreated control (media only) control (mean ± SEM, *n* = 4). Effects relative to vehicle control (0.25% DMSO; black bar) were determined by one-way ANOVA with post-hoc Dunnett, where p < 0.05 is significant. *p ≤ 0.05, ***p ≤ 0.001.

A highly drug-resistant cancer cell line, HCT-116, was selected for bioactivity screening assays due to the high expression levels of proteins involved in chemo-resistance, including Nrf2^34,35^. The 11 hit compounds were tested for their effect on the viability of human colon carcinoma cells following 24 h treatment at 2.5 µM, using a cell-based MTS assay (Fig. 2A). Selecting a low concentration to identify the most potent drugs reduces the likelihood of the identification of off-target effects in downstream investigations, as well as avoiding background effects from DMSO on cell viability and *Nrf2* expression. Cell viability was reduced following treatment with compounds 73735 and 354844, and therefore excluded from gene expression experiments. The effect of the remaining ligands on *Nrf2* mRNA expression was determined 4 h after treatment, alongside quercetin as a positive control (Fig. 2B). Quercetin has been shown to bind both quadruplexes and duplex DNA^36–38^, and is well known to upregulate *Nrf2* in vitro^39,40^. As expected, quercetin at 25 µM significantly upregulated *Nrf2* expression compared to the vehicle control (1.33-fold, *p* = 0.01)^41^. Gene expression of *Nrf2* was also found to increase in response to compound 300289 (1.51-fold, *p* ≤ 0.001) and was increased, though not significantly in response to 2 further ligands (compound 143491 (1.29-fold, *p* = 0.09) and compound 317003 (1.25-fold, *p* = 0.17)). *Nrf2* expression was significantly decreased following treatment with compound 202386 (0.8-fold, p ≤ 0.001). The Nrf2 pathway is involved in drug metabolism in the cell and is upregulated in response to certain chemopreventive agents^42^, so it was anticipated that the presence of some of these ligands would activate this pathway. Compound 300289 is pinafide, a member of the naphthalimide family, derivatives of which have known DNA-binding and anti-tumour properties^43^.

Although a plethora of ligands that upregulate Nrf2 protein expression have been identified, there are relatively few that successfully inhibit its expression^6^. It has been demonstrated that the knockdown of *Nrf2* using miRNA in patients with acute myeloid leukaemia reduced tumour cell clonogenicity and improved their chemotherapeutic responsiveness^44^ and it is therefore interesting that we have identified one ligand that appears to reduce *Nrf2* gene expression (compound 202386, *p* ≤ 0.001). Although many ligands have been shown to disrupt the interaction of Nrf2/Keap1^6^, this is the first example of controlling the promoter of *Nrf2* directly. The upregulation of *Nrf2* supresses oxidative stress and inflammatory responses and increases cell survival^4^, which is an important mechanism of cell defence in diseases driven by low-level, chronic inflammation, including cancer^45^. Much focus has been given to modulating post-translational mechanisms, such as the Keap1-Nrf2 interaction and subsequent expression of antioxidant genes under *Nrf2* regulation using small molecules, such as dimethyl fumarate (DMF), which has been used for several decades for the management of psoriasis and, more recently, relapsing-remitting multiple sclerosis^1^. Many more Nrf2-modulating drugs are currently in phase III testing, however, there are issues regarding specificity and off-target effects. Relatively few studies demonstrate regulation of *Nrf2* gene expression directly, so it remains to be seen whether this mechanism of action eases or exacerbates off-target effects.

### Characterisation of Nrf-2 modulating compounds by CD melting

**Fig. 3:**
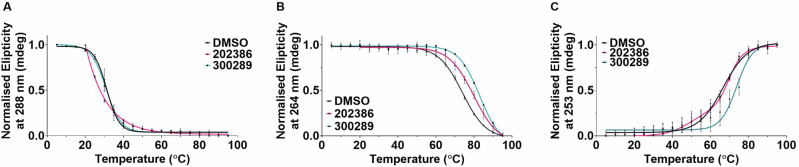
DNA melting experiments in the presence of ligands. Normalised Circular dichroism melting experiment for Nrf2C (**A**), Nrf2G (**B**), and DS (**C**) DNA in presence of 5 molar equivalents of DMSO control (black) NSC 202386 (pink) and NSC 300289 (teal) in 10 mM sodium cacodylate, 100 mM potassium chloride, pH 6.5. Data are presented as Mean ± SD, *n* = 2.

To determine the comparative interaction between different types of DNA structures, compound 300289 and compound 202386 were subjected to further binding measurements using CD melting. We measured the melting of C-rich Nrf2C and its complementary G-rich sequence Nrf2G (5’-GGG-AAG-GGA-GCA-AGG-GCG-GGA-GGG-3’), and control double-stranded sequences (5’-GGC-ATA-GTG-CGT-GGG-CGT-TAG-C-3’ plus complement). DNAs were annealed at 10 µM in 10 mM NaCaco, 100 mM KCl at pH 6.5. CD melting experiments was performed in the presence of 5 molar equivalents of ligand (50 μM) or the equivalent amount of DMSO without ligand as a control. Example CD melting experiments can be found in Fig. 3 and Δ*T*_m_ values can be found in Table 2, further supporting experiments are detailed in Supplementary Fig. S3.

**Table 2:** Stabilisation temperatures measured by CD melting

Structure	Compound	Δ*T*_m_ (°C)		
		Nrf2C	Nrf2G	DS
	202386	-12 ± 1.0 **	+5 ± 1.0***	0 ± 0.7
	300289	-1 ± 1.0	+9 ± 0.3***	+9 ± 0.8***

10 μM DNA annealed in 10 mM sodium cacodylate, 100 mM potassium chloride at pH 6.5. Values represent the average of two experiments and the errors are standard deviations.

Compound 300289 was found to have no significant effect on the stability of Nrf2C (Δ*T*_m_ = −1 ± 1.0 °C), but was found to stabilise both Nrf2G (Δ*T*_m_ = +9 ± 0.3 °C, *p* ≤ 0.001) and double stranded DNA (Δ*T*_m_ = +9 ± 0.8 °C, *p* ≤ 0.001). Compound 202386 was found to have a significant destabilisation effect on Nrf2C (Δ*T*_m_ -12 ± 1.0 °C, *p* ≤ 0.01), weak stabilisation of Nrf2G (Δ*T*_m_ = +5 ± 0.3 °C, *p* ≤ 0.001) and no effect on double stranded DNA (Δ*T*_m_ = 0 ± 0.7 °C). The destabilisation effects of 202386 were complicated by poor ligand solubility at low temperatures, so measurements were only able to start from 20 °C, as opposed to from 5 °C. The quoted Δ*T*_m_ is therefore an underestimate. It is important to note here that destabilisation potentially indicates that ligand binding stabilises a different/alternative structure to the original conformation. This could be another i-motif in the equilibrium mixture^46^, which is less stable than the main annealed population, an alternative partially folded species^47^, hairpin^48^ or the single strand^49^. There are a number of compounds that destabilise i-motif structures, most of which also strongly stabilise G-quadruplex DNA^11,12,49^. In this case, we observe an example of a ligand that very strongly destabilises i-motif DNA, but with only weak stabilisation of G-quadruplex DNA.

### Determination of binding and specificity of Nrf-2 modulating compounds

Given the stabilising and biological properties of both 202386 and 300289, we were interested in determining their binding affinities against the i-motif and G-quadruplex forming sequences from Nrf-2. Dissociation constants (*K*_**d**_s) were determined using UV titrations and are depicted in Supplementary Figs. S4–6 and summarised in Table 3. We determined the *K*_**d**_ of 202386 to be 0.25 ± 0.1 µM against Nrf2C (Supplementary Figs. S4A and S5A) and 0.6 ± 0.1 µM against Nrf2G (Supplementary Figs. S4B and S5B), demonstrating a two-fold preference for the Nrf-2 i-motif over the G-quadruplex. Ligand 202386 was also found to bind double helical DNA with much lower affinity (*K*_**d**_ = 1.9 ± 0.1 µM, Supplementary Figs. S4A and S5G), showing a six-fold preference for the i-motif. The *K*_**d**_ for 202386 against Nrf2C (250 nM) is in the same range as BisA and Pyridostatin binding the i-motif forming sequence from the human telomere (*K*_**d**_ = 220 nM and 320 nM respectively)^50^ when measured at the same pH (6.5). Other literature ligands with similar binding affinities are PBP1 (*K*_**d**_ = 300 nM, measured at pH 6 against Bcl2)^51^ and compound 1 (*K*_**d**_ = 200 nM, measured at pH 6 against Hras-1)^52^. To the best of our knowledge, the only ligands with tighter binding to i-motifs are IF2 and TmPyP4 (*K*_**d**_ = 34.6 nM and 28.7 nM respectively, measured at pH 7.4 against the *BmPOUM2* i-motif)^53^. In contrast to the tight binding of 202386, ligand 300289 showed comparatively weaker binding to Nrf2C (*K*_**d**_ = 20 ± 1.8 µM, Supplementary Figs. S4C and S6A) and Nrf2G (*K*_**d**_ = 6.6 ± 0.9 µM, Supplementary Figs. S4D and S6B), with a three-fold preference for the G-quadruplex forming sequence from *Nrf2*.

**Table 3:** Dissociation constants determined by UV titrations

Structure	Compound	*K*_d_ (µM)						
		Nrf2C	hTeloC	DAPC	Nrf2G	hTeloG	DAPG	DS
	202386	0.25 ± 0.1	1.1 ± 0.2	0.9 ± 0.1	0.6 ± 0.1	1.0 ± 0.1	1.6 ± 0.2	1.9 ± 0.1
	300289	20 ± 1.8	10 ± 1.3	6.0 ± 0.9	6.6 ± 0.9	6.2 ± 0.4	8.2 ± 0.7	2.4 ± 0.2

10 μM DNA annealed in 10 mM sodium cacodylate, 100 mM potassium chloride at pH 6.5. Values represent the average of three experiments and the errors are standard deviations.

To explore specificity against other i-motif and G-quadruplex forming sequences in the genome, we examined binding against sequences from the human telomere (hTeloC: 5’-TAA-CCC-TAA-CCC-TAA-CCC-TAA-CCC-3’ and hTeloG: 5’-GGG-TTA-GGG-TTA-GGG-TTAG-GGT-TA-3’) and the promoter region of death associated protein (DAPC: 5’-CCC-CCG-CCC-CCG-CCC-CCG-CCC-CCG-CCC-CC-3’ and DAPG: 5’-GGG-GGC-GGG-GGC-GGG-GGC-GGG-GGC-GGG-GG-3’). From this, we found that 300289 binds all the G-quadruplexes with approximately similar affinity (*K*_**d**_ = 6.6 ± 0.9 µM, 6.2 ± 0.4 µM and 8.2 ± 0.7 µM for Nrf2G, hTeloG and DAPG respectively, Supplementary Figs. S4D and S6B, D, E) and was more specific for the DAPC i-motif (*K*_**d**_ = 6.0 ± 0.9 µM, Supplementary Figs. S4C and S6A) and hTeloC (*K*_**d**_ = 10. ± 1.3 µM, Supplementary Figs. S4C and S6C) compared to Nrf2C (*K*_**d**_ = 20 ± 1.8 µM). 202386 was also found to bind the other i-motifs (*K*_**d**_ = 1.1 ± 0.2 µM for hTeloC and *K*_**d**_ = 0.9 ± 0.1 µM for DAPC) (Supplementary Figs. S4A, S6A and C). However, 202386 was found to bind the i-motif from *Nrf2*, better than all other DNAs examined (*K*_**d**_ = 250 nM), with between two to six-fold preference for the *Nrf2* i-motif over the other structures. This preference for binding the *Nrf2* i-motif complements its destabilisation properties.

Ligand 202386, as part of one of the NCI libraries, has previously been shown to have weak Anti-HIV-1 integrase activity^54^. However, previous screening against RNA-Protein interactions within the HIV-1 lifecycle^55^ and the human telomeric G-quadruplex^56^ did not raise this ligand as a hit. Others who have examined this compound against *Entamoeba histolytica*^57^ and *Mycobacterium tuberculosis*^58^ also did not find it to be a hit. Given the binding and destabilisation properties of 202386 against the i-motif forming sequence from *Nrf2* and the corresponding effects on *Nrf2* gene expression, this opens up the possibility of further compounds being developed to target *Nrf2* by binding and destabilising i-motif DNA. In this case, ligand 202386 is a promising lead for development for higher affinity analogues, with improved general solubility and also selectivity for i-motif over other non-canonical structures. As it is available from the NCI, it also may be a useful probe for further i-motif binding studies.

## Conclusions

We have characterised a previously unexplored i-motif DNA structure in the promoter region of *Nrf2* and have determined that it is able to form at near physiological conditions. Further, we have identified a number of ligands that bind to the i-motif structure in the *Nrf2* promoter and two that can modulate *Nrf2* gene expression. Ligand 202386 was found to bind the i-motif from *Nrf2* very tightly (250 nM) with two to six-fold preference for the i-motif from *Nrf2* over other i-motifs, G-quadruplexes and double-stranded DNA. This is the first example of controlling the promoter of *Nrf2* by targeting DNA structures and offers a potential alternative mode of action for targeting this gene. The hit compounds could be used as tools for modulation of *Nrf2* or as leads for the development of novel future therapies for restoring cellular homoeostasis and the efficacy of current cancer therapies.

## Methods

### General experimental

Oligonucleotides, purified by reverse phase HPLC, were purchased from Eurogentec. The nucleotide sequence for *Nrf2* was 5’-CCC-TCC-CGC-CCT-TGC-TCC-CTT-CCC-3’ DNA stock solutions were dissolved in MilliQ water and diluted to the appropriate concentration in 10 mM sodium cacodylate, 100 mM potassium chloride at the specified pH and annealed by heating at 95 °C for 5 min and cooling to room temperature overnight. A compound library of 1584 compounds (Diversity Set IV) was obtained from the National Cancer Institute (NCI) Developmental Therapeutics Program (DTP) and was constituted to 1 mM in DMSO. Quercetin (Sigma Aldrich) was solubilised to 10 mM in DMSO and is utilised in experiments due to known duplex and quadruplex DNA binding effects and upregulation of *Nrf2*, in vitro^39,40,59^.

### Circular dichroism spectroscopy and melts

Nrf2C (10 µM) was annealed in buffer at pH 4.0 to pH 8.0 to give a total volume of 200 µL for each sample. Spectra were recorded using a Jasco J-810 spectropolarimeter with a Starna Scientific type-21 quartz cuvette of 1 mm path length. Scans were performed at 20 °C from 200 nm to 320 nm with a scanning speed of 200 nm/min, a response time of 1 s, a pitch of 0.5 nm, and a bandwidth of 2 nm. Controls containing only the respective buffer were recorded and subtracted from the experimental data. All spectra represent an average of four scans. The transitional pH (pH_*T*_) was calculated by the Bolzmann sigmoidal curve fitting of the ellipticity signal at 288 nm and respective pH values.

Furthermore, CD melts were recorded on a Jasco J-1500 spectropolarimeter using a 1 mm path-length quartz cuvette. C-rich *Nrf2* and its complementary G-rich *Nrf2* (5’-GGG-AAG-GGA-GCA-AGG-GCG-GGA-GGG-3’), and control double-stranded sequences (5’-GGC-ATA-GTG-CGT-GGG-CGT-TAG-C-3’ plus complement) were prepared at 10 µM each in 10 mM sodium cacodylate, 100 mM potassium chloride at pH 6.5. CD melt was performed in the presence of 5 molar equivalents of ligand (50 μM) or DMSO and a spectrum from 230 nm – 320 nm at a speed of 200 nm/min was measured with increasing temperature at a rate of 1 °C/min from 5 to 95 °C. Four accumulations of scans are recorded at 5 °C intervals, 1 s response time, 0.5 nm pitch, 2 nm bandwidth. The ellipticity for Nrf2C (at 288 nm), Nrf2G (at 264 nm), and double-stranded DNA (at 253 nm) was plotted against temperature and fitted with the Bolzmann sigmoidal curve to determine the melting temperature (T_m_) at which 50% of the thermal denaturation had taken place by using Graphpad Prism (version 9).

### UV spectroscopy

All UV experiments were performed on a Jasco V760 UV-vis spectrophotometer. Thermal melting curves were derived from absorbance recordings at 295 nm. Samples were held at 4 °C for 5 min then heated to 95 °C three times at a rate of 0.25 °C/min and data was recorded every 0.1 °C during melting and annealing. *T*_*M*_ values were determined using first derivative methods. Thermal difference spectra were obtained by conducting a wavelength scan between 320 nm and 230 nm at 4 °C and 95 °C. The spectra of the folded structure at 4 °C was subtracted from that of the unfolded structure at 95 °C. The data were normalised and the maximum change in absorption was set to +1, as previously described^25^.

UV-based binding assays were determined using the wavelength at maximum absorbance of NSC 202386 at 320 nm and NSC 300289 at 335 nm in 10 mM sodium cacodylate, 100 mM potassium chloride at pH 6.5. DNA was annealed in the same buffer and added in concentrations up to 50 µM. DNA samples used were Nrf2C [5’-CCC-TCC-CGC-CCT-TGC-TCC-CTT-CCC-3’], hTeloC [5’-TAA-CCC-TAA-CCC-TAA-CCC-TAA-CCC-3’], DAPC [5’-CCC-CCG-CCC-CCG-CCC-CCG-CCC-CCG-CCC-CC-3’] and DS [5’-GGC-ATA-GTG-CGT-GGG-CGT-TAG-C-3’] and Nrf2G [5’-GGG-TTA-GGG-TTA-GGG-TTAG-GGT-TA-3’], hTeloG [GGGTTAGGGTTAGGGTTAGGGTTA], DAPG [5’-GGG-GGC-GGG-GGC-GGG-GGC-GGG-GGC-GGG-GG-3’]. Experiments were performed in triplicate. Data shown as Mean ± SD, n = 3.

### Nuclear Magnetic Resonance (NMR)

^1^H NMR spectra of *Nrf2* sequence were recorded at 298 K using an 800 MHz Bruker Avance III spectrometer equipped with a triple resonance z-gradient probe^10,60^. To minimise saturation of labile proton signals, jump return and watergate solvent suppression pulse sequences were used. Samples were prepared to a final concentration of 10 µM in buffer (pH 6.5). 10% D_2_O was added immediately before commencing experiments. NMR spectra were acquired and processed using Bruker’s Top- Spin™ software package (v3.1.7 Bruker Biospin).

### Fluorescent Indicator Displacement (FID) assay

*Nrf2*-binding ligands were identified from a library of 1584 compounds using a fluorescent indicator displacement (FID) assay^33^. The fluorescent indicator thiazole orange (TO) was diluted to 1 µM in pH 6.5 buffer and the Nrf2C sequence was annealed at 50 µM DNA concentration in pH 6.5 buffer. TO buffer was excited at 430 nm and the background fluorescence was recorded between 450 nm and 650 nm using a BMG LabTech Omega plate reader. The background fluorescence at 450 nm was normalised as 0%. Nrf2C was then added to each well to a concentration of 1 µM, mixed, and incubated for 10 min at room temperature. Fluorescence emission intensity at 450 nm was normalised as 100%. Ligands were then added, in triplicate, to a final concentration of 2.5 µM. To determine DC_50_ values, ligands were titrated with spectra measured between each addition. Displacement was calculated as previously described^33^_._ Where *D*_x_ is TO displacement resulting from substitution with the candidate ligand and *D*_x_ = 1 – (*F*_x_/*F*_0_), *F*_0_ is the fluorescence intensity of the TO-i-motif complex in the absence of any ligands and *F*_x_ is the fluorescence intensity after titration with the candidate ligand.

### MTS assay

Human colon carcinoma cells (HCT-116; purchased directly from ATCC (CCL-247™), UK and were used as directed without authentication or further testing) were cultured in DMEM containing 10% FBS and 1 ⨯ penicillin/streptomycin (Gibco, UK) and plated at a cell density of 0.02 × 10^6^ cells/well onto 96-well flat bottom plates (Greiner Bio-one, UK) 24 h prior to experiment commencement. On the day of assay, media was replaced with 100 µL fresh medium supplemented with 2.5 µM of each ligand, 0.25% DMSO (vehicle control), or media only (untreated). After 24 h, 10 µL of MTS assay reagent (CellTiter 96® AqueousOne Solution Cell Proliferation Assay; Promega, UK) was added and cells were further incubated for 4 h at 37 °C, 5% CO_2_, in a humidified atmosphere. Absorbance at 490 nm was read using a BMG Omega plate reader. Cell viability was determined as a percentage of absorbance against cells treated with media only (untreated). A concentration of 2.5 µM was selected based on the relatively high concentration of DMSO in the final solutions, 0.25% DMSO did not influence growth effects or cell death within 24 h.

### qRT-PCR

HCT-116 were cultured as described above and plated at a cell density of 0.2 × 10^6^ cells/well onto 24-well plates (Greiner Bio-one, UK) 24 h prior to experiment commencement. On the day of the assay, media was replaced with 1 mL of supplemented media containing 2.5 µM ligand, 0.25% DMSO (vehicle control), 25 µM quercetin (positive control), or media only (untreated control). Cells were incubated for 4 h at 37 °C, 5% CO_2_, in a humidified atmosphere. Media was then removed, and the cells were lysed, RNA collected, and reverse transcribed to cDNA, using a FastLane Cell cDNA Kit (Qiagen, UK) according to the manufacturer’s instructions. 25 ng cDNA from each reaction was utilised for each qRT-PCR reaction, using primers prepared and validated by PrimerDesign, UK (*Nrf2* forward primer: 5’-CCCAGCACATCCAGTCAGA-3’; reverse primer, 3’-CAGTCATCAAATACAAAGCATCT-3’), and PrecisionPlus qPCR Master Mix (without ROX). The following PCR conditions were used utilising a QIAgen RotorGene Q-series PCR machine: activation at 95 °C for 2 min, followed by 50 cycles of DNA denaturation (10 s, 95 °C), DNA annealing and data collection (60 s, 60 °C). Ct values were normalised against a stable reference gene GAPDH (Primer Design, UK) using the 2^-ΔΔCT^ method^61^ and presented as the fold change against cells treated with media only (untreated). Quercetin was used as a positive control due to its known activity on *Nrf2* gene expression^39^.

### Reporting summary

Further information on research design is available in the Nature Portfolio Reporting Summary linked to this article.

## Supplementary information

Supplementary Information

Reporting Summary

## Data Availability

All data generated in this study are available within the Article, Supplementary Information and can be downloaded from 10.5281/zenodo.13924302.
