# Supplementary material for: Modulation of Nrf2 expression by targeting i-motif DNA
Source: Commun Chem. 2025 Jan 6;8:5. doi: 10.1038/s42004-024-01387-w (PMC11704350; doi:10.1038/s42004-024-01387-w)
Supplement: Supplementary file 1 — Supplementary Information [file 42004_2024_1387_MOESM1_ESM.pdf]

Modulation of Nrf2 expression by targeting i-motif DNA

E.F. Warner,[1] D. Guneri,[2] M. A. O'Connell,[1] C.J. MacDonald,[3] Z.A.E Waller[2]*

[1]School of Pharmacy, University of East Anglia, Norwich, Norfolk, NR4 7TJ, United Kingdom.

[2] UCL School of Pharmacy, London, WC1N 1AX, United Kingdom. [3]School of Chemistry; University of East Anglia, Norwich, Norfolk, NR4 7TJ, United Kingdom.

*Corresponding author. Email: z.waller@ucl.ac.uk

Supplementary Figures and Tables

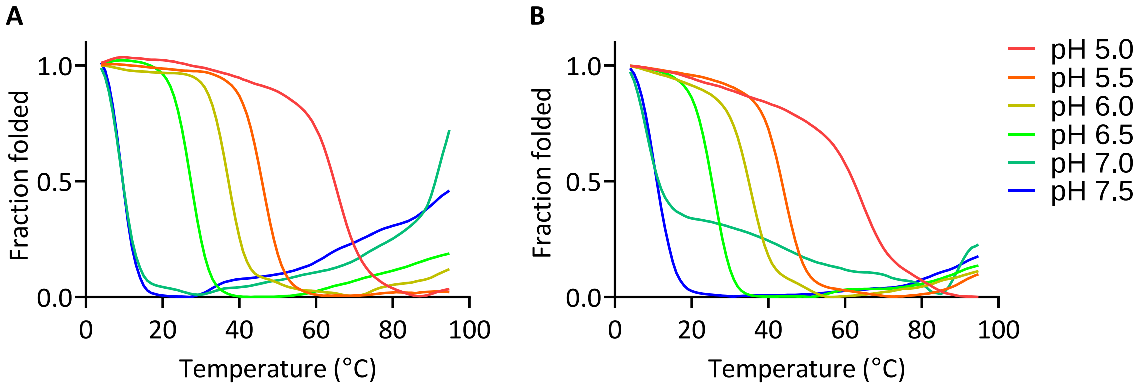

Supplementary Figure S1: UV Melting and Annealing of Nrf2C. Representative normalised UV melting **(A)** and annealing curves **(B)** measured at 295 nm of Nrf2C (5 μM DNA) in buffer containing 10 mM sodium cacodylate, 100 mM potassium chloride at pH 5.0-7.5.

Supplementary Table S1: UV melting and annealing temperatures for Nrf2C. (2.5 μM DNA) in buffer containing 10 mM sodium cacodylate, 100 mM potassium chloride at pH 5.0-7.5. Data representative of the mean of 3 consecutive melt/anneal cycles ± SD.

pH	T_M (°C)	T_A (°C)
5.0	66 ± 0.0	64. ± 0.1
5.5	46 ± 0.6	43 ± 0.6
6.0	37 ± 0.0	35 ± 0.0
6.5	27 ± 0.4	26 ± 0.2
7.0	10 ± 0.6	8 ± 0.4
7.5	12 ± 1.8	13 ± 2.1

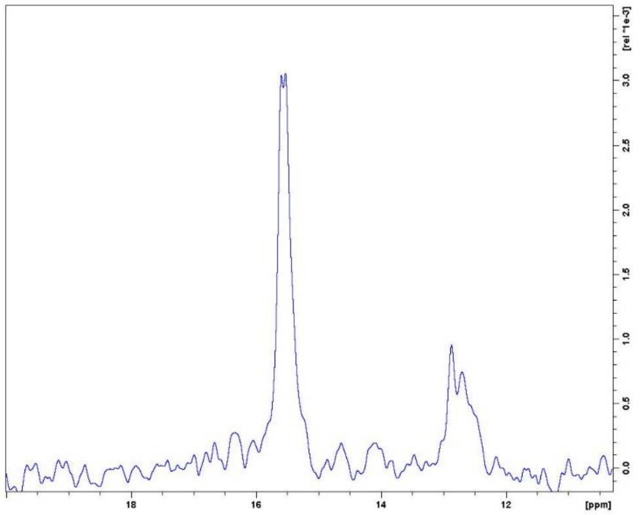

Supplementary Figure S2: NMR Spectrum of Nrf2C. Imino proton region of the ^{1}H NMR spectra (at 298 K) of Nrf2C (10 µM DNA) in buffer containing 10 mM sodium cacodylate, 100 mM potassium chloride, with 10% D_2O, at pH 6.5.

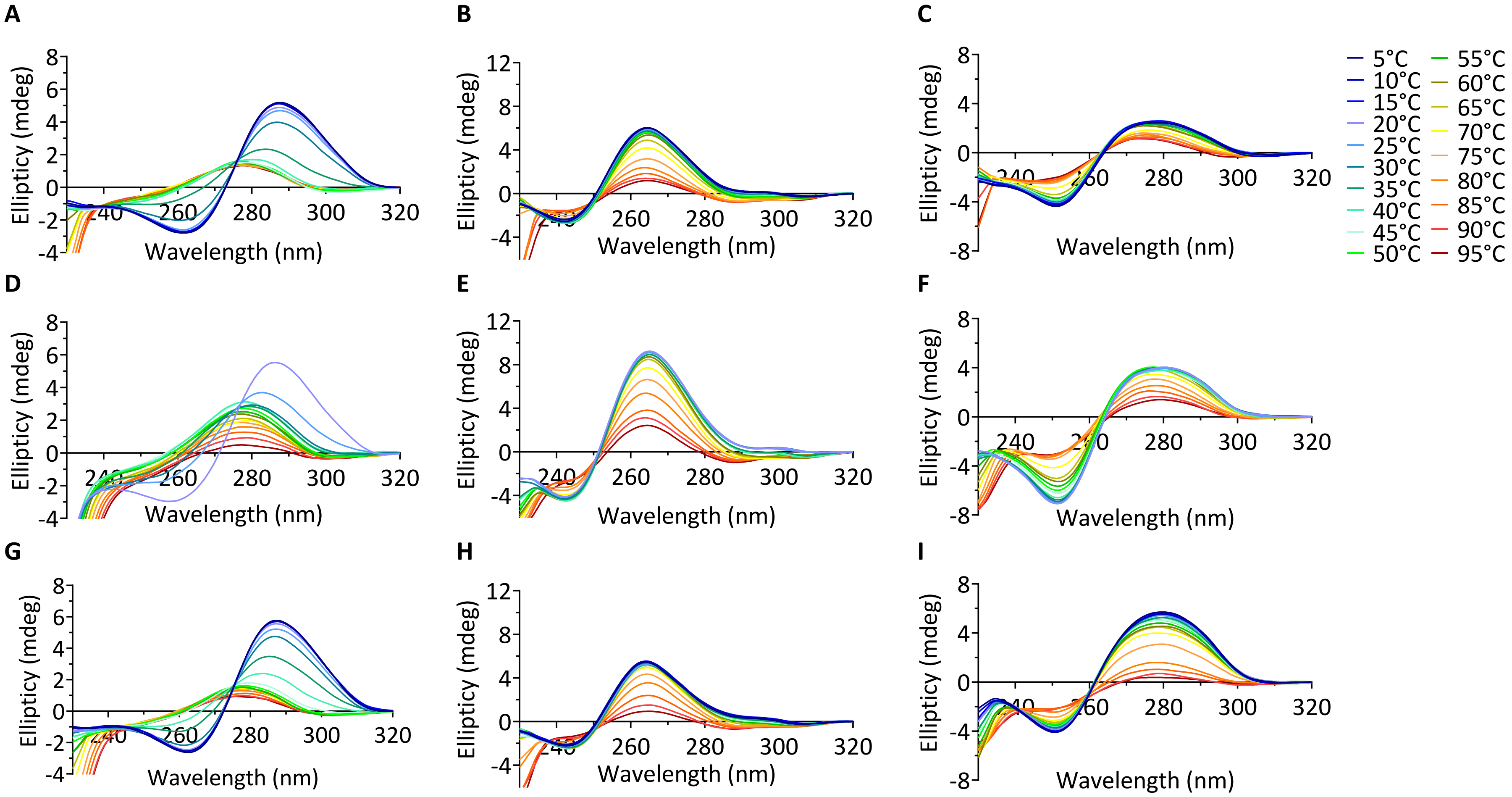

Supplementary Figure S3: CD melting experiments with ligands. 10 µM DNA in 10 mM sodium cacodylate, 100 mM potassium chloride at pH 6.5 **(A, D, G)** Nrf2C, **(B, E, H)** Nrf2G, and **(C, F, I)** Double stranded DNA supplemented with 5 molar equivalent of **(A, B, C)** DMSO, **(D, E, F)** NSC 202386, **(G, H, I)** NSC 300289. DNA samples in DMSO and NSC 300289 were melted from 5°C to 95°C while NSC 202386 was melted from 20°C to 95°C due to solubility issues.

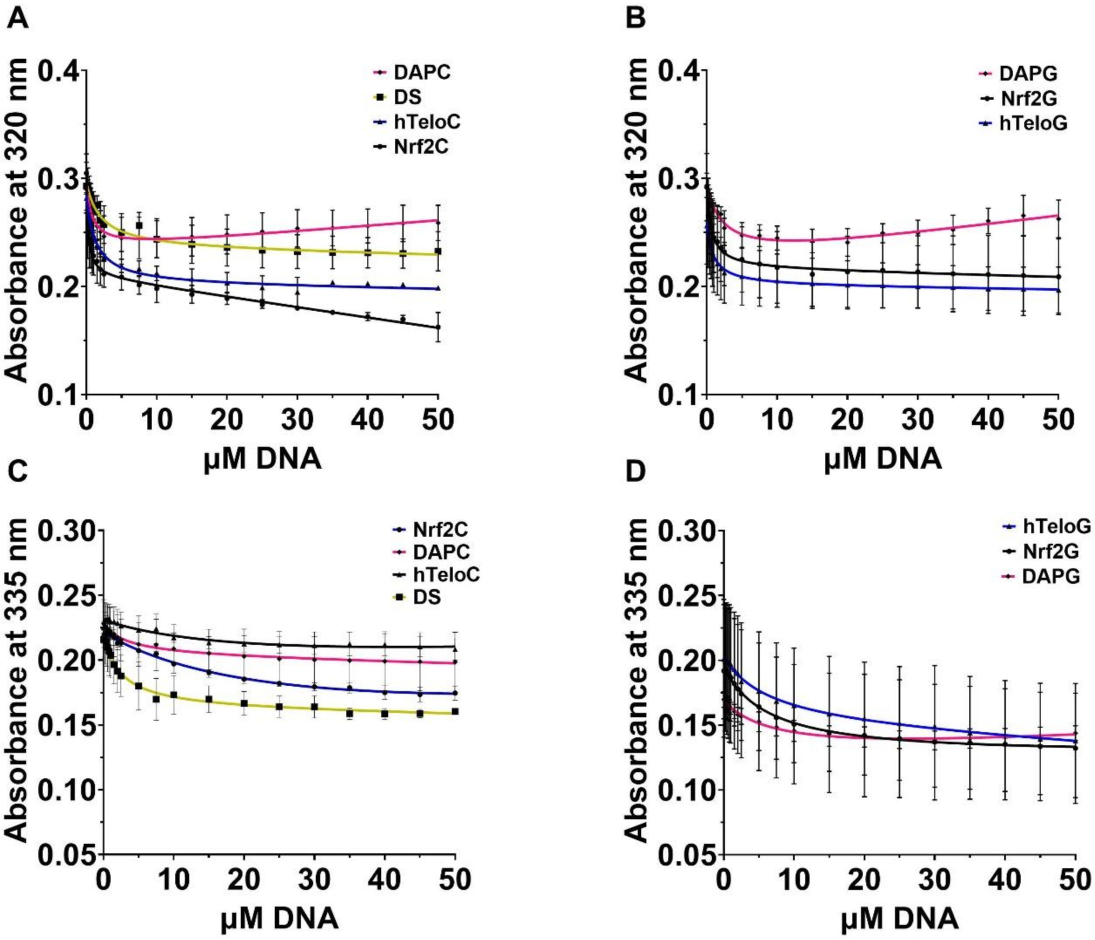

Supplementary Figure S4: UV Binding Experiments. UV binding assay showing absorbance at absorbance maximum of NSC 202386 (A,B) at 320 nm and NSC 300289 at 335 nm (C,D) in 10 mM sodium cacodylate, 100 mM potassium chloride at pH 6.5. DNA was annealed in the same buffer and added as 0-50 µM Nrf2C, hTeloC, DAPC and DS (A,C) and Nrf2G, hTeloG, DAPG (B,D). Data shown as Mean ± SD, n=3.

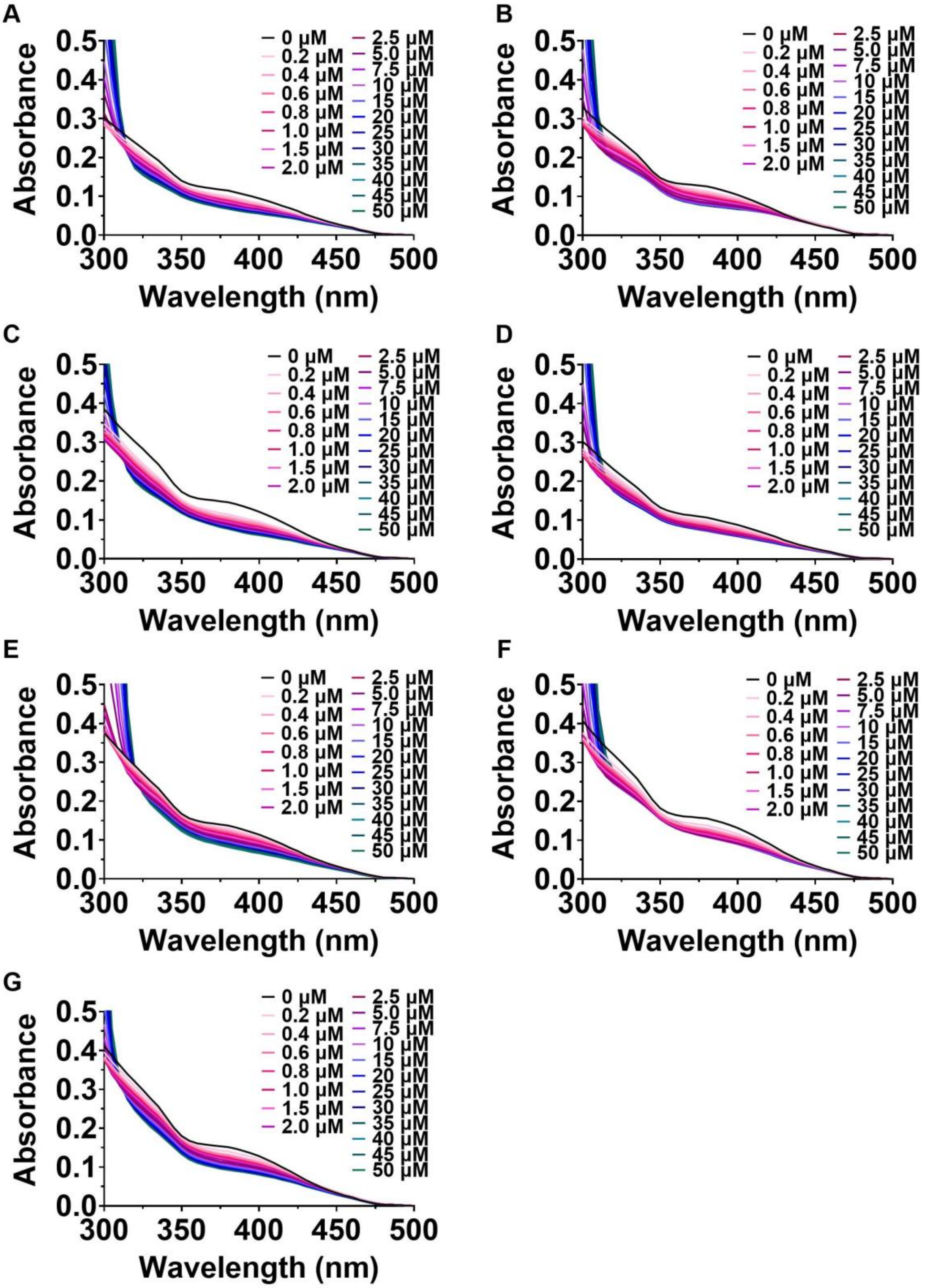

Supplementary Figure S5. UV binding assay showing absorbance spectrum from 300 nm to 500 nm of NSC 202386 in 10 mM sodium cacodylate, 100 mM potassium chloride at pH 6.5. DNA was annealed in the same buffer and added as 0-50 µM Nrf2C (A), hTeloC (C), DAPC (E) Nrf2G (B), hTeloG (D), DAPG (F) and DS (G). Data shown example with spectrum baseline corrected to 500 nm.

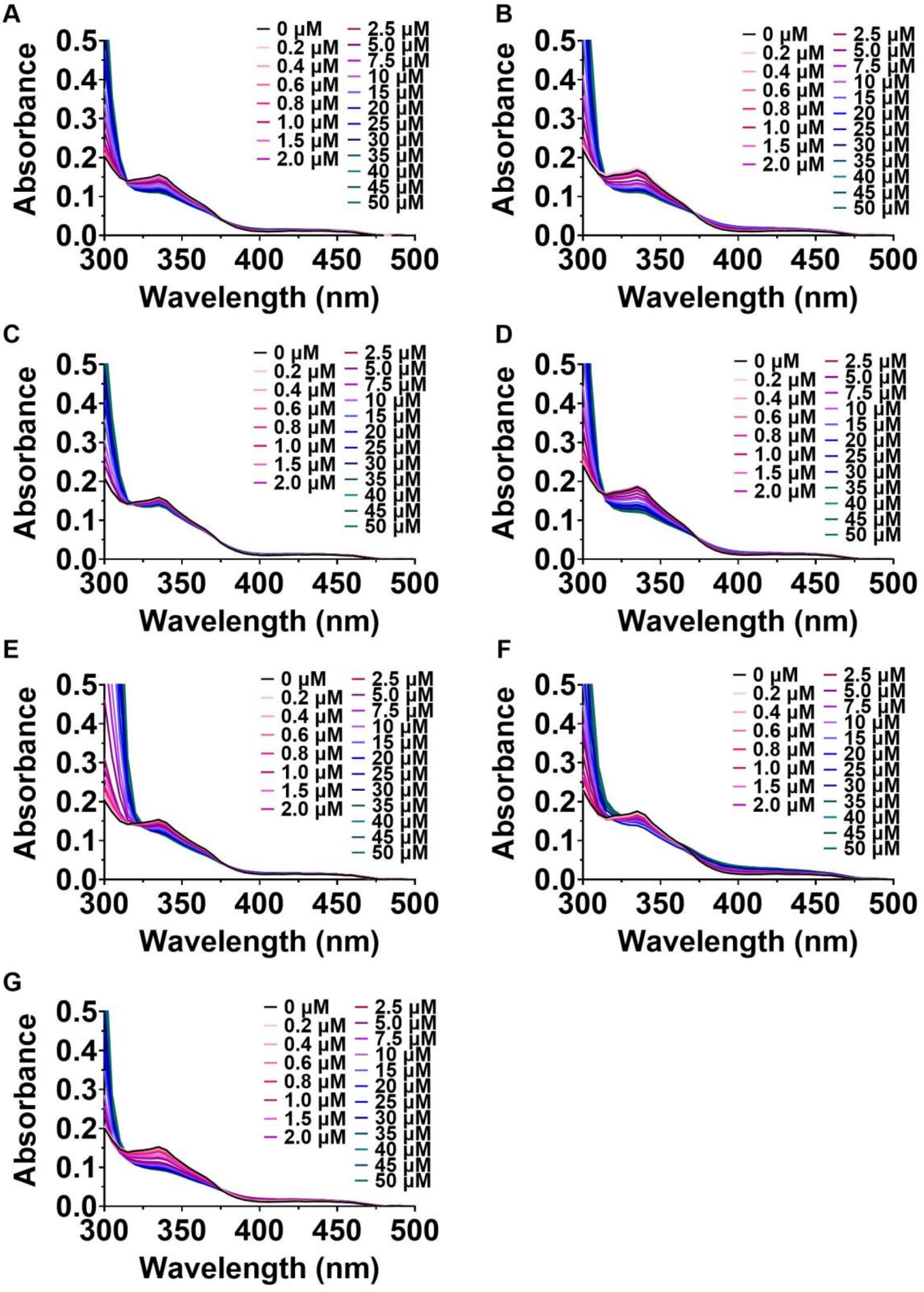

Supplementary Figure S6. UV binding assay showing absorbance spectrum from 300 nm to 500 nm of NSC 300289 in 10 mM sodium cacodylate, 100 mM potassium chloride at pH 6.5. DNA was annealed in the same buffer and added as 0-50 μM Nrf2C (A), hTeloC (C), DAPC (E) Nrf2G (B), hTeloG (D), DAPG (F) and DS (G). Data shown example with spectrum baseline corrected to 500 nm.
